# The rate, cost and outcomes of parathyroidectomy in the united states dialysis population from 2016–2018

**DOI:** 10.1186/s12882-022-02848-x

**Published:** 2022-06-21

**Authors:** Mark D Danese, Kathleen M Fox, Jennifer L. Duryea, Pooja Desai, Robert J Rubin

**Affiliations:** 1 Outcomes Insights, Inc., 30200 Agoura Road, Suite 230, Agoura Hills, CA 91301 USA; 2 Global Health Economics, Amgen, Inc., Thousand Oaks, CA USA; 3 Janssen, Titusville, NJ USA; 4 Division of Nephrology and Hypertension, Georgetown University, Washington, DC USA

**Keywords:** Mineral and bone disorder (MBD), Secondary hyperparathyroidism (SHPT), Parathyroidectomy, Costs, Outcomes, End-stage kidney disease (ESKD)

## Abstract

**Background:**

In end-stage kidney disease, patients may undergo parathyroidectomy if secondary hyperparathyroidism cannot be managed medically. This study was designed to estimate the parathyroidectomy rate in the United States (US) and to quantify changes in costs and other outcomes after parathyroidectomy.

**Methods:**

This was a retrospective observational cohort study using US Renal Data System data for 2015–2018. Parathyroidectomy rates were estimated for adult hemodialysis and peritoneal dialysis patients alive at the beginning of 2016, 2017, and 2018 who were followed for a year or until parathyroidectomy, death, or transplant. Incremental differences in economic and clinical outcomes were compared before and after parathyroidectomy in adult hemodialysis and peritoneal dialysis patients who received a parathyroidectomy in 2016 and 2017.

**Results:**

The rate of parathyroidectomy per 1,000 person-years decreased from 6.5 (95% CI 6.2-6.8) in 2016 to 5.3 (95% CI 5.0-5.6) in 2018. The incremental increase in 12-month cost after versus before parathyroidectomy was $25,314 (95% CI $23,777-$27,078). By the second month after parathyroidectomy, 58% of patients had a corrected calcium level < 8.5 mg/dL. In the year after parathyroidectomy (versus before), hospitalizations increased by 1.4 per person-year (95% CI 1.3-1.5), hospital days increased by 12.1 per person-year (95% CI 11.2-13.0), dialysis visits decreased by 5.2 per person-year (95% CI 4.4-5.9), and office visits declined by 1.3 per person-year (95% CI 1.0-1.5). The incremental rate per 1,000 person years for hematoma/bleed was 224.4 (95% CI 152.5-303.1), for vocal cord paralysis was 124.6 (95% CI 59.1-232.1), and for seroma was 27.4 (95% CI 0.4-59.0).

**Conclusions:**

Parathyroidectomy was a relatively uncommon event in the hemodialysis and peritoneal dialysis populations. The incremental cost of parathyroidectomy was mostly attributable to the cost of the parathyroidectomy hospitalization. Hypocalcemia occurred in over half of patients, and calcium and phosphate levels were reduced. Clinicians, payers, and patients should understand the potential clinical and economic outcomes when considering parathyroidectomy.

**Supplementary Information:**

The online version contains supplementary material available at 10.1186/s12882-022-02848-x.

## Background

Patients with end-stage kidney disease (ESKD) often have secondary hyperparathyroidism (SHPT) which is characterized by elevated parathyroid hormone (PTH) in response to kidney failure. SHPT is commonly associated with mineral and bone disorder (MBD) which includes associated effects on serum levels of calcium (Ca) and phosphate (P) [1].Typically, SHPT and MBD are managed through the use of vitamin D, phosphate binders, and/or calcimimetics [2]. However, patients who cannot be managed with currently available options may undergo a parathyroidectomy to reduce PTH and facilitate the management of Ca and P [1, 3].

Parathyroidectomy is relatively uncommon. The rate of parathyroidectomy was estimated at 5.4 per 1,000 person-years using United States (US) inpatient data from 2002 through 2011 and was relatively stable through the latter part of the time period [4]. Rates were highest in younger patients (age 20–44 years) and were higher in women than men, and inpatient mortality decreased from 1.7 to 0.8% from 2002 to 2011. A separate study using 2013 national readmission data showed that approximately 17.2% of patients with SHPT had an unplanned hospital re-admission within 30 days of a parathyroidectomy [5]. National parathyroidectomy rates or readmission rates for the US have not been published since these investigations. A study of 181 patients who underwent parathyroidectomy between January 1, 2008 and December 31, 2010 showed that the monthly physician charges increased from $1,531 in the 6 months before parathyroidectomy to $1,931 in the 6 months afterward [6]. The authors also showed that physician encounters increased after parathyroidectomy, and that 31% of patients had a diagnosis code for hypocalcemia after parathyroidectomy. However, the study was limited by its small sample size and lack of inpatient data.

Therefore, the goals of this study were to provide more current estimates of the parathyroidectomy rate in adult, US hemodialysis (HD) and peritoneal dialysis (PD) patients, and to quantify changes in costs as well as key outcome measures after parathyroidectomy, including subsequent parathyroidectomy, mortality, surgical side effects, dialysis visits, office visits, hospitalizations, and selected laboratory values.

## Materials and Methods

Please see Supplementary Materials for more details on the methods used in this study.

### Study Design

This was a retrospective observational cohort study using USRDS data from January 1, 2015 through December 31, 2018 [7]. Specific details of the files used are available in the USRDS Researcher’s Guide [8]. The study protocol was reviewed by the Advarra Institutional Review Board (IRB) and received an exemption determination on April 2, 2021.

### Study Observation Period

For the parathyroidectomy rate analyses, the study index date was January 1 of each year from 2016–2018. The baseline period was the 3-month period prior to January 1 and follow-up ended on December 31 of each year. For the parathyroidectomy cost and outcomes analyses, the baseline period was the 12-month period prior to parathyroidectomy; follow-up began with the parathyroidectomy and ended on December 31, 2018.

For all analyses, follow-up was truncated at the following events: end of continuous Medicare Part A, B, and D enrollment, kidney transplant, death, or the end of the data (December 31, 2018). For the parathyroidectomy rate analyses, follow-up was also truncated at parathyroidectomy.

### Study Populations

For the parathyroidectomy rate analyses, we included all prevalent HD and PD patients age 18 years or older as of the first day of each year from 2016 through 2018. Patients must have been continuously enrolled in Medicare Part A and Part B coverage with Medicare as the primary payer and must not have received a parathyroidectomy during the baseline period.

For the parathyroidectomy cost and outcomes analyses, the study population was all adult HD and PD patients who underwent parathyroidectomy between January 1, 2016 and December 31, 2017 who were at least 19 years old at the time of parathyroidectomy and received either HD or PD (but not both) during the entire 12-month baseline period. Patients receiving HD must have received at least 21 HD visits over the previous 3 months (i.e., an average of at least 7 of the expected 13 dialysis sessions each month). Patients must have had Medicare Part A, Part B, and Part D coverage during the entire baseline period. We excluded patients who had a prior parathyroidectomy during the observation period (i.e., as early as January 1, 2015) as well as patients with a diagnosis code for primary hyperparathyroidism who had no diagnosis of secondary hyperparathyroidism.

### Baseline Variables

For parathyroidectomy rate estimation, the key baseline variables were age, sex, dialysis modality, and ESKD network. For the parathyroidectomy cost and outcomes cohort, additional baseline variables included time since dialysis initiation (vintage), cause of ESKD, and comorbid conditions. Comorbid conditions were based on the Charlson Comorbidity Index, excluding renal disease [9]. Phosphate binder, calcimimetics, and intravenous (IV) vitamin D were identified from Part B and Part D claims. Oral vitamin D was not available for 2015 or 2016 and was not reported.

### Outcome Variables

For parathyroidectomy rate estimation, the outcome was based on the parathyroidectomy date of service. Costs were defined as Medicare payment and patient contracted amounts (copayment, coinsurance, and deductible) for all covered services, adjusted to 2020 using the full-year 2020 medical component of the Consumer Price Index. Resource utilization included dialysis visits, hospitalization, hospitalization days, and office visits. Surgical side effects were based on diagnosis codes for vocal cord paralysis or laryngeal nerve injury, hematoma/bleeding related to surgery, wound infection related to surgery, and seroma formation related to surgery (Supplementary Materials Table 1) [6, 10]. Laboratory measures for corrected Ca (cCa) and P were based on CROWNWeb data (PTH was not available). We excluded laboratory values in the month of parathyroidectomy because only the month and year were known and therefore “before” and “after” parathyroidectomy could not be determined. We also identified hypocalcemia (cCa < 8.5 mg/dL) and severe hypocalcemia (cCa < 7.5 mg/dL) each month [11, 12]. Finally, we identified subsequent parathyroidectomy procedures after the index surgery, and all-cause mortality.

### Analyses

Rates were estimated assuming a Poisson distribution for the variance [13]. Analyses of the parathyroidectomy rate were stratified by the following factors: modality (HD versus PD), year (2016–2018), vintage group (0–2, 2–5, and 5 + years since dialysis initiation), ESKD network, and race. We also combined the results for all years in a Poisson regression model. Unadjusted cumulative costs were estimated over the 12-month baseline and follow-up periods (the parathyroidectomy procedure was included in the follow-up period). Costs were partitioned by month using inverse probability of censoring weights to account for patients who were lost to follow-up [14, 15]. We estimated the difference in the 12-month cost estimates and in rates of resource utilization before and after parathyroidectomy. We used bootstrapping to estimate 95% confidence intervals. Analyses related to cost were conducted overall as well as for HD and PD patients separately. We conducted a sensitivity analysis of parathyroidectomy cost using complete cases, defined as patients alive and uncensored through the end of month 12 after parathyroidectomy. Surgical complication rates were estimated as the difference between the cumulative 90-day rates before and after parathyroidectomy, with confidence intervals estimated using bootstrapping.

Laboratory data for cCa and P were reported as monthly means. Hypocalcemia was summarized as a proportion per month.

The raw data were converted to a standardized format (Generalized Data Model [16]). Jigsaw software (Outcomes Insights, Inc., Agoura Hills, CA) was used to extract all data for analysis. The final analysis dataset was created in R (version 3.6.3) [17]. All statistical analyses were performed using R.

## Results

### Parathyroidectomy Rate

**Fig. 1:**
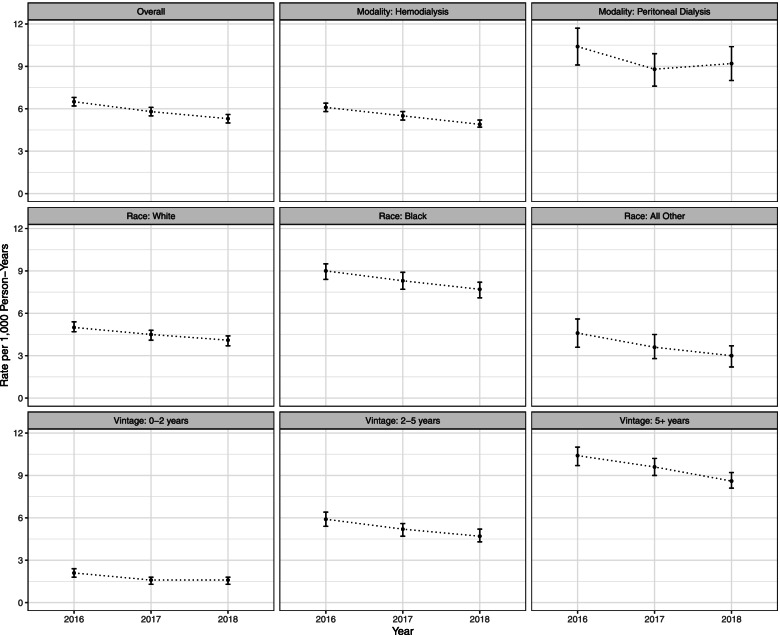
Parathyroidectomy Rates by Year Overall and by Dialysis Modality, Race, and Dialysis Vintage Subgroups

The rate of parathyroidectomy per 1,000 person-years decreased during the 3 years of observation in this study from 6.5 (95% CI 6.2 to 6.8) in 2016 to 5.8 (95% CI 5.5 to 6.1) in 2017 to 5.3 (95% CI 5.0 to 5.6) in 2018. Over this 3-year time period, the annual rate ranged from 4.9 to 6.1 in HD patients and from 8.8 to 10.4 in PD patients. Over the time period, the highest rate in any stratum was for patients younger than 40 years of age, which ranged from 19.3 to 21.9. Detailed results by year and by modality, race, and vintage are provided in Fig. 1 and additional detail in Supplementary Materials Tables 2–4. In an adjusted Poisson regression model across all years, dialysis modality, age, dialysis vintage, race, and year were all statistically significantly associated with the rate of parathyroidectomy (Supplementary Materials Table 5). In particular, PD patients were 1.7-fold (95% CI 1.6 to 1.8) more likely to undergo parathyroidectomy than HD patients when conditioning on the other variables. Patients on dialysis for 5 or more years were 4.4-fold (95% CI 3.9 to 4.9) more likely to undergo parathyroidectomy compared to patients on dialysis for less than 2 years. 

### Parathyroidectomy Cost and Outcomes

The process of creating the cohort is described in Supplementary Materials Table 6. There were 11,014 patients who had a parathyroidectomy. After application of the study inclusion and exclusion criteria, there were 3,008 patients in the final cohort, of whom 259 were PD patients. Baseline demographics of the overall cohort as well as the HD and PD sub-cohorts are provided in Table 1. In the overall cohort, the mean age at parathyroidectomy was 51.7 years, 49.4% were male, and 56.1% were Black. The most common causes of ESKD were hypertension (37.1%) and diabetes (27.3%). Within the 90-day period prior to parathyroidectomy, 42.8% received a calcimimetic, 46.8% received IV vitamin D, and 47.1% received a phosphate binder. In the overall cohort, the average duration of follow-up was 20.7 months, with 16.6% of the cohort dying, 11.6% having a kidney transplant, and 4.8% having a subsequent parathyroidectomy procedure by December 31, 2018.

**Table 1:** Population characteristics for all parathyroidectomy patients by dialysis modality

Variable	All (*N* = 3,008)	HD (*N* = 2,749)	PD (*N* = 259)
	*Mean (SD) or Count (%)*	*Mean (SD) or Count (%)*	*Mean (SD) or Count (%)*
**Age at Parathyroidectomy**			
Age (years)	51.7 (13.1)	51.8 (13.0)	50.4 (13.9)
Dialysis Vintage (years)	7.0 (3.9)	7.1 (3.9)	5.3 (3.4)
**Sex and Race Categories**			
Male (%)	1,487 (49.4%)	1,395 (50.7%)	92 (35.5%)
White (%)	1,173 (39.0%)	1,048 (38.1%)	125 (48.3%)
Black (%)	1,687 (56.1%)	1,567 (57.0%)	120 (46.3%)
Asian (%)	73 (2.4%)	NR	NR
Other (%)	75 (2.5%)	NR	NR
**Cause of ESKD**			
Hypertension (%)	1,116 (37.1%)	1,007 (36.6%)	109 (42.1%)
Diabetes (%)	822 (27.3%)	789 (28.7%)	33 (12.7%)
Glomerulonephritis (%)	525 (17.5%)	467 (17.0%)	58 (22.4%)
Cystic kidney (%)	172 (5.7%)	NR	NR
Other (%)	75 (2.5%)	NR	NR
**Year of Parathyroidectomy**			
2016 (%)	1,594 (53.0%)	1,462 (53.2%)	132 (51.0%)
2017 (%)	1,414 (47.0%)	1,287 (46.8%)	127 (49.0%)
**Other Baseline Variables**			
Hospitalization in Prior 12 Months (%)	1,431 (47.6%)	1,361 (49.5%)	70 (27.0%)
Serum Calcium (corrected, mg/dL)	9.4 (0.8)	9.4 (0.8)	9.6 (0.9)
Serum Phosphate (mg/dL)	6.4 (1.6)	6.4 (1.6)	6.5 (1.7)
Calcimimetic Use Within 90 Days (%)	1,288 (42.8%)	1,205 (43.8%)	83 (32.0%)
IV Vitamin D Use Within 90 Days (%)	1,407 (46.8%)	NR	NR
Phosphate binder Use Within 90 Days (%)	1,417 (47.1%)	1,312 (47.7%)	105 (40.5%)
**Charlson Comorbidity Index**			
CCI Index (continuous)	3.6 (3.4)	3.7 (3.4)	1.9 (2.5)
CCI Score 0 (%)	504 (16.8%)	405 (14.7%)	99 (38.2%)
CCI Myocardial Infarction (%)	450 (15.0%)	432 (15.7%)	18 (6.9%)
CCI Congestive Heart Failure (%)	1,279 (42.5%)	1,231 (44.8%)	48 (18.5%)
CCI Peripheral Vascular Disease (%)	840 (27.9%)	820 (29.8%)	20 (7.7%)
CCI Cerebrovascular Disease (%)	361 (12.0%)	341 (12.4%)	20 (7.7%)

Selected cardiovascular-related CCI conditions included in table. *NR* = counts < 11 not reportable according to the data use agreement with the USRDS

*CCI *Charlson Comorbidity Index, *HD *hemodialysis, *PD *peritoneal dialysis, *SD *standard deviation, *IV *intravenous

**Fig. 2:**
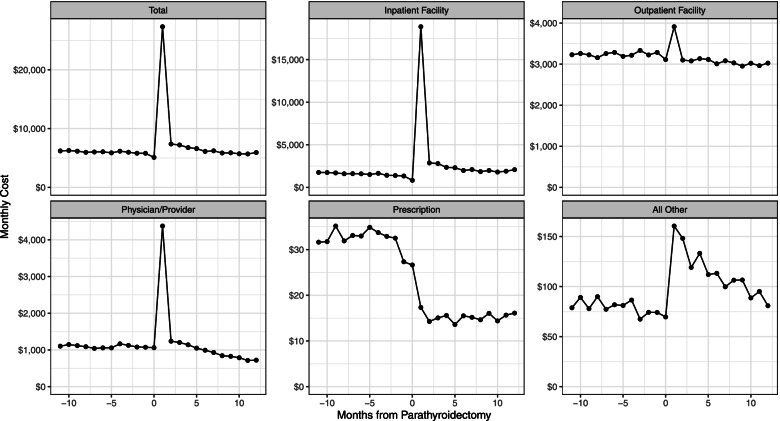
Healthcare Costs by Type for the 12 Months Before and After Parathyroidectomy. Note: Costs include all payer and patient paid amounts. Prescription costs include only Medicare Part D costs. All costs are inflated to 2020 dollars. The cost of parathyroidectomy surgery is included in month 1

The incremental difference in the paid amount (rounded to the nearest dollar including payer and patient amounts) between the year after and the year before parathyroidectomy was $25,314 (95% CI $23,777 to $27,078; Table 2). Of this, the patient portion was approximately 7.5% ($1,887). The largest single component of the incremental difference was related to inpatient facility payments (primarily for the parathyroidectomy procedure itself) which were $24,758 (95% CI $23,492 to $26,340). Incremental 12-month outpatient facility payments were lower after parathyroidectomy: -$1,358 (95% CI -$910 to -$1,747). The incremental 12-month physician/provider payments were higher after parathyroidectomy: $1,701 (95% CI $1,258 to $2,228). The results were similar using the subset of patients who were alive and observable 12 months after parathyroidectomy (N = 2,581; Supplementary Materials Table 7). Monthly costs over the entire 24-month interval for all services combined and by type of service are shown in Fig. 2. The total costs stratified by dialysis modality showed similar patterns to the overall cohort (Table 2; Supplementary Materials Fig. 1).

**Table 2:** Costs Before and After Parathyroidectomy, Overall and by Dialysis Modality

Cost Type	Before	After	Difference	Lower CI	Upper CI
***Overall***					
Inpatient Facility	$18,121	$42,878	$24,758	$23,492	$26,340
Outpatient Facility	$38,780	$37,421	-$1,358	-$1,747	-$910
Physician/Provider	$13,127	$14,828	$1,701	$1,258	$2,228
Prescription	$384	$183	-$201	-$224	-$177
All Other	$948	$1,364	$415	$308	$521
Total	$71,360	$96,675	$25,314	$23,777	$27,078
***Hemodialysis (HD)***					
Inpatient Facility	$19,437	$43,315	$23,877	$22,570	$25,453
Outpatient Facility	$39,097	$37,738	-$1,359	-$1,766	-$875
Physician/Provider	$13,789	$15,126	$1,337	$873	$1,882
Prescription	$384	$182	-$203	-$227	-$177
All Other	$1,005	$1,388	$383	$265	$493
Total	$73,713	$97,749	$24,037	$22,492	$25,827
***Peritoneal Dialysis (PD)***					
Inpatient Facility	$4,144	$38,245	$34,102	$28,825	$38,573
Outpatient Facility	$35,412	$34,058	-$1,354	-$2,607	-$43
Physician/Provider	$6,103	$11,660	$5,557	$4,233	$6,641
Prescription	$386	$202	-$183	-$258	-$94
All Other	$347	$1,103	$757	$441	$1,051
Total	$46,391	$85,268	$38,878	$32,357	$44,492

Note: Costs include all payer and patient paid amounts. Prescription costs include only Medicare Part D costs. All costs are inflated to 2020 dollars. Confidence intervals estimated using bootstrapping. “Before” and “After” reflect the mean cost for the 12-month periods before and after parathyroidectomy. The cost of parathyroidectomy is included in the “after” interval

*HD *hemodialysis, *PD *peritoneal dialysis

Mortality and subsequent parathyroidectomy were highest in the 30-day period immediately after the parathyroidectomy (Table 3). The initial 30-day mortality rate per 1,000 person-years was 167.6 (95% CI 116.3 to 218.9). Afterward the rate varied between 76 and 102, depending on the time interval. The initial 30-day parathyroidectomy rate per 1,000 person-years was 156.9 (95% CI 107.0 to 206.8). The rate declined to between 19.1 and 46.8 over the course of the first year after parathyroidectomy and was 12.9 (95% CI 8.0 to 17.5) thereafter.

**Table 3:** Death rate and subsequent parathyroidectomy rate per 1,000 person-years over time after parathyroidectomy

Days After Index Parathyroidectomy	Patients at Risk	Patients with Event	Person Years at Risk	Event Rate	Lower CI	Upper CI
***Death***						
1—30	3,008	41	245	167.6	116.3	218.9
31—90	2,954	37	480	77.1	52.3	102.0
91—180	2,888	71	699	101.6	78.0	125.3
181—360	2,787	100	1,324	75.5	60.7	90.3
361—1094	2,581	251	2,451	102.4	89.7	115.1
***Subsequent parathyroidectomy***						
1—30	3,008	38	242	156.9	107.0	206.8
31—90	2,916	NR	NR	19.1	6.6	31.5
91—180	2,843	32	684	46.8	30.6	63.0
181—360	2,711	36	1,280	28.1	18.9	37.3
361—1094	2,478	30	2,323	12.9	8.3	17.5

*CI *confidence interval, *NR *counts < 11 not reportable according to the data use agreement with the USRDS

The rate of side effects potentially related to parathyroidectomy varied by the specific event. The incremental difference (90 days after minus 90 days before) in the rate per 1,000 person years for hematoma or bleed was 224.4 (95% CI 152.5 to 303.1). The incremental rate for vocal cord paralysis was 124.6 (95% CI 59.1 to 232.1). The incremental rate for seroma was much lower, at 27.4 (95% CI 0.4 to 59.0). We identified no wound infections in the cohort.

The incremental differences in the utilization rate per person-year (after parathyroidectomy minus before) for office visits, dialysis visits, and hospitalizations are provided in Table 4. Hospitalizations increased by 1.4 (95% CI 1.3 to 1.5), hospital days increased by 12.1 (95% CI 11.2 to 13.0), dialysis visits decreased by 5.2 (95% CI 4.4 to 5.9), and office visits declined by 1.3 (95% CI 1.0 to 1.5). The monthly rates of hospitalizations, dialysis visits, and office visits before and after parathyroidectomy are included in Supplementary Materials Figs. 2–4.

**Table 4:** 12-Month Healthcare Utilization Rates and 90-day Surgical Side Effect Rates

Outcome	Before PTx	After PTx	Difference	Lower CI	Upper CI
***Utilization rate (per person-year)***					
Physician office visit	10.6	9.3	-1.3	-1.5	-1.0
Dialysis visit	147.7	142.5	-5.2	-5.9	-4.4
Hospitalization	1.2	2.5	1.4	1.3	1.5
Hospitalization days	6.7	18.8	12.1	11.2	13.0
***Surgical side effect rate (per 1,000 person-years)***					
Hematoma or bleed	14.7	239.0	224.4	152.5	303.1
Vocal cord paralysis	13.3	138.0	124.6	59.1	232.1
Seroma	5.3	32.8	27.4	0.4	59.0
Wound infection	0.0	0.0	0.0	0.0	0.0

Note: “Before” and “After” refer to the 12-month periods before and after parathyroidectomy (PTx) for utilization, and the 90-day periods before and after PTx for surgical side effects. *CI* = confidence interval

**Fig. 3:**
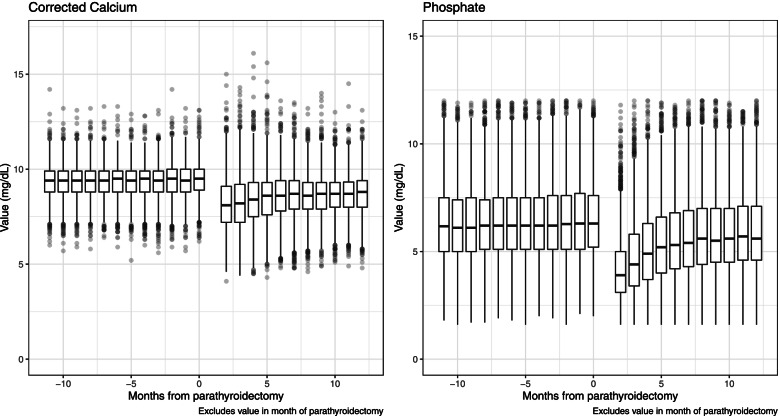
Boxplots of Corrected Calcium and Phosphate for the 12 Months Before and After Parathyroidectomy. Note: The median is shown as a horizontal line within each box. The edges of each box represent the 25% and 75% percentile (interquartile range). Vertical lines (“whiskers”) represent 1.5 times the interquartile range. Values outside of this range are shown as dots whose intensity is proportional to the number of dots to minimize overplotting

**Fig. 4:**
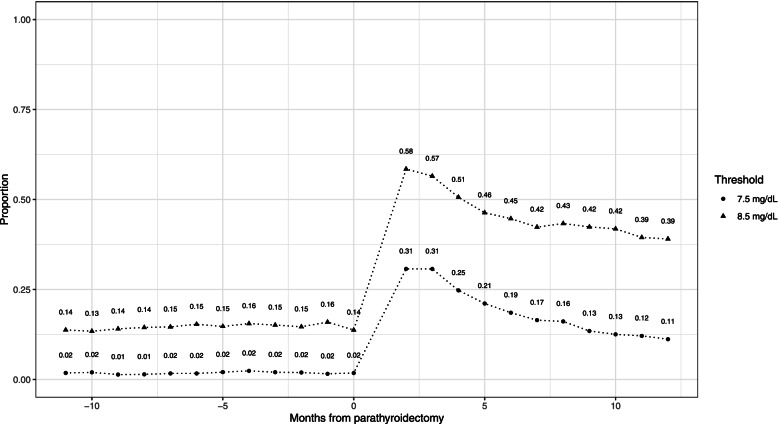
Proportion of Patients with Hypocalcemia for the 12 Months Before and After Parathyroidectomy. Note: Hypocalcemia is based on a corrected calcium threshold of < 8.5 mg/dL and severe hypocalcemia is based on a threshold of < 7.5 mg/dL

Monthly median cCa and P were stable in the 12 months prior to parathyroidectomy but varied over time after parathyroidectomy (Fig. 3). The rate of hypocalcemia (cCa < 8.5 mg/dL) increased from approximately 14–16% of patients per month before parathyroidectomy to 58% by the second month after parathyroidectomy (Fig. 4). Thereafter, it declined to 39% by month 12. The trend was similar for severe hypocalcemia, ranging from 1 to 2% per month before parathyroidectomy to 31% for months 2 and 3 after parathyroidectomy, declining to 11% by month 12 (Fig. 4).

## Discussion

This study presents the most current parathyroidectomy rates to date and is the first to provide detailed cost, utilization and laboratory information for the year before and after parathyroidectomy. Parathyroidectomy remains a relatively uncommon event for dialysis patients. The rate of parathyroidectomy per 1,000 person years declined from 6.5 to 5.3 between 2016 and 2018; however, it is difficult to extrapolate long-term trends from 3 years of data. These rates are slightly higher than the overall rate of 5.4 per 1,000 estimated by Kim et al. using annual inpatient data from 2002 to 2011 [4]. Kim et al. used inpatient data that included transplant patients (who we excluded) and could not exclude multiple parathyroidectomy hospitalizations from the same person. In contrast, we required Medicare Part A, B, and D coverage and Kim et al. estimated rates across all payers. Despite these differences, our finding that the parathyroidectomy rate is higher in younger patients is consistent with Kim, et al., and we extend these findings to show that parathyroidectomy is extremely rare in the oldest patients. We also show that there is a 1.7-fold higher parathyroidectomy rate in PD patients compared to HD patients after accounting for age, race, and dialysis vintage.

Patients undergoing parathyroidectomy cost the healthcare system an incremental $25,000, the majority of which is from inpatient facility payments for the parathyroidectomy itself but which also includes the higher rate of hospitalization and hospitalization days during the 12 months afterward. The incremental cost of parathyroidectomy was even higher in PD patients ($39,000). In terms of other consequences of parathyroidectomy, both subsequent parathyroidectomy and all-cause mortality were at their highest levels during the 30-day period after the parathyroidectomy. The higher mortality after surgery is consistent with the findings from Kestenbaum, et al., although the absolute rate in that study (386 per 1,000 person-years) is more than twice the rate in our study [18].  Since the Kestenbaum study was based on patients undergoing parathyroidectomy prior to 2000, the documented decline in the inpatient mortality rate from 2002 to 2011 [4] suggests that the difference in rates between our study and that by Kestenbaum et al. are related to improvements in post-operative survival for patients over time.

Belozeroff et al. showed that hypocalcemia was the most common surgical complication, followed by bleeding, and that wound infection and seroma were rare [6]. Our study provides more precise estimates of these events, correcting for background rates that might lead to an overestimate in a dialysis population. Also, we used serum corrected calcium instead of diagnosis codes to identify hypocalcemia and showed that hypocalcemia is almost twice as common than shown by Belozeroff et al. (31%), with 58% of patients having cCa < 8.5 mg/dL and 31% having cCa < 7.5 mg/dL in month 2 after parathyroidectomy. These results are consistent with the overall trends in cCa and P which were stable for the year before parathyroidectomy, declined after surgery, and then rose gradually over the course of the following year to a mean level that was still lower than the pre-surgery values. These findings suggest that clinicians should be aware of the signs and symptoms of hypocalcemia in these patients, some of whom may have hypocalcemia for a year after surgery.

We also showed that inpatient hospitalizations increased by 1.4 per person per year and hospital days increased by 12.1 days per person per year. Given that outpatient HD occurs on 3 out of 7 days (43%), one would expect exactly 5.2 fewer dialysis visits with 12.1 more days in the hospital. Hence, the increase in hospitalizations likely explains the lower rate of dialysis visits.

Patients who undergo parathyroidectomy are a select subgroup of patients with SHPT. At the time of parathyroidectomy in our 2016–2017 parathyroidectomy cohort, 47% of patients were receiving phosphate binders, 47% were receiving IV vitamin D, and 43% were receiving calcimimetics. In comparison, in the overall ESKD population in quarter 1 of 2017, 63% of patients were receiving phosphate binders, 42% were receiving IV vitamin D, and 30% were receiving calcimimetics [7]. Hence, the differences in the proportions were such that the parathyroidectomy population had 13% more patients receiving calcimimetics, a 16% fewer patients receiving phosphate binders, and 5% more patients receiving IV vitamin D. This suggests that patients undergoing parathyroidectomy may be more likely to be using calcimimetics and IV vitamin D, and less likely to be using phosphate binders. However, given that phosphate binder use may be under-reported compared to other sources, these results should be interpreted with caution [19].

This study is strengthened by its broad coverage of US dialysis patients, the availability of detailed laboratory information, and the availability of comprehensive Medicare claims data that include direct health care expenditures across all settings of care (inpatient, outpatient, pharmacy, and home care). As with any study using observational data, there are limitations and other factors to be considered when interpreting these findings. Our focus was on HD and PD patients who had Medicare as their primary payer. Our rates were estimated for 2016–2018, and our parathyroidectomy cohort was limited to patients who had a parathyroidectomy in 2016–2017. When estimating the annual parathyroidectomy rates, it is likely that some procedures were not the “first-ever” parathyroidectomy for some patients. However, because we excluded patients who had a parathyroidectomy within the 3 months prior to each year, we minimized the potential for double-counting parathyroidectomy episodes. Also, we were not able to evaluate whether changes in the parathyroidectomy rate were associated with changes in vitamin D or calcimimetics use because oral medication data were only available in patients with Medicare Part D coverage. Finally, we note that these results apply to the US and that in other regions of the world parathyroidectomy utilization and practice patterns may be different.

The cohorts created in this study only included patients with Medicare fee-for-service coverage. However, since commercially insured patients initiating dialysis are covered by their primary private insurer during a 36-month coordination of care period and are not included in these data, these results may not apply to the full dialysis population. As a result, in both the parathyroidectomy rate and cost estimation analyses, there is a risk that younger patients in our study may be different from the total under-65 years of age population initiating dialysis. Also, because different kinds of costs including medications are reimbursed as part of the bundled dialysis payment, we could not separate all medication costs from medical costs. Similarly, costs by type may vary depending on the specific details of the bundled payments for a particular year. As a result, cost increases for medications used to manage patients after parathyroidectomy (e.g., for hungry bone syndrome) cannot be enumerated in this study. Also, to the extent that parathyroidectomy might reduce the risk and associated cost of long-term outcomes beyond one year, these analyses could not capture the associated cost reductions. Finally, we did not have parathyroid hormone levels in the data, which would have been useful for characterizing the short-term and long-term effectiveness of parathyroidectomy.

In conclusion, parathyroidectomy is a relatively uncommon event that continues to occur at a rate of about 5.3 to 6.5 per 1,000 person-years. The incremental 12-month cost of parathyroidectomy is approximately $25,000, and the most common complication of surgery is the risk of hypocalcemia which occurs in more than half of patients. Both mean cCa and P decline after surgery and, although they rise during the following year, they remain lower than the presurgical values. Because of the implications of parathyroidectomy for clinicians, payers and patients, the USRDS should consider reporting parathyroidectomy-related metrics in its annual report. This would facilitate clear communication of the clinical and economic outcomes associated with parathyroidectomy.

## Supplementary Information

**Additional file 1: Supplemental Table 1.**  Diagnosis codes for surgical side effects. **Supplemental Table 2.**  Rate of Parathyroidectomy Overall and Within Strata: 2016. **Supplemental Table 3.**  Rate of Parathyroidectomy Overall and Within Strata: 2017. **Supplemental Table 4.** Rate of Parathyroidectomy Overall and Within Strata: 2018. **Supplemental Table 5.** Poisson Regression Model of Factors Associated with Parathyroidectomy. **Supplemental Table 6.** Parathyroidectomy Cohort Attrition Table13. **Supplemental Table 7.**  Costs Before and After Parathyroidectomy, Overall and by Dialysis Modality:  Sensitivity Analysis. **Supplemental Figure 1.** Total Payments by Type (All Patients Using Censoring Weights). **Supplemental Figure 2.** Office Visit Utilization Over Time **Supplemental Figure 3.** Hemodialysis Visits Over Time. **Supplemental Figure 4.** Hospitalizations Over Time

## Data Availability

The data that support the findings of this study are available from the USRDS, but restrictions apply to the availability of these data, which were used under license for the current study, and so are not publicly available.
