# Supplementary material for: The rate, cost and outcomes of parathyroidectomy in the united states dialysis population from 2016–2018
Source: BMC Nephrol. 2022 Jun 21;23:220. doi: 10.1186/s12882-022-02848-x (PMC9215010; doi:10.1186/s12882-022-02848-x)
Supplement: Supplementary file 1 — Additional file 1: Supplemental Table 1. Diagnosis codes for surgical side effects. Supplemental Table 2. Rate of Parathyroidectomy Overall and Within Strata: 2016. Supplemental Table 3. Rate of Parathyroidectomy Overall and Within Strata: 2017. Supplemental Table 4. Rate of Parathyroidectomy Overall and Within Strata: 2018. Supplemental Table 5. Poisson Regression Model of Factors Associated with Parathyroidectomy. Supplemental Table 6. Parathyroidectomy Cohort Attrition Table13. Supplemental Table 7. Costs Before and After Parathyroidectomy, Overall and by Dialysis Modality: Sensitivity Analysis. Supplemental Figure 1. Total Payments by Type (All Patients Using Censoring Weights). Supplemental Figure 2. Office Visit Utilization Over Time Supplemental Figure 3. Hemodialysis Visits Over Time. Supplemental Figure 4. Hospitalizations Over Time [file 12882_2022_2848_MOESM1_ESM.docx]

Supplementary Materials

**The Rate, Cost and Outcomes of Parathyroidectomy in the United States Dialysis Population from 2016-2018**

Table of Contents

[1. Methods 3](#_Toc91586566)

[1.1 Study Design 3](#_Toc91586567)

[1.2 Study Observation Period 3](#_Toc91586568)

[1.3 Study Populations 3](#_Toc91586569)

[1.4 Baseline Variables 4](#_Toc91586570)

[1.5 Outcome Variables 5](#_Toc91586571)

[1.6 Analyses 5](#_Toc91586572)

[2. Tables 7](#_Toc91586573)

[3. Figures 21](#_Toc91586574)

**Tables and Figures**

[Supplemental Table 1: Diagnosis codes for surgical side effects 3](#_Toc91579672)

[Supplemental Table 2: Rate of Parathyroidectomy Overall and Within Strata: 2016 6](#_Toc91579673)

[Supplemental Table 3: Rate of Parathyroidectomy Overall and Within Strata: 2017 8](#_Toc91579674)

[Supplemental Table 4: Rate of Parathyroidectomy Overall and Within Strata: 2018 10](#_Toc91579675)

[Supplemental Table 5: Poisson Regression Model of Factors Associated with Parathyroidectomy 12](#_Toc91579676)

[Supplemental Table 6: Parathyroidectomy Cohort Attrition Table 13](#_Toc91579677)

[Supplemental Table 7: Costs Before and After Parathyroidectomy, Overall and by Dialysis Modality: Sensitivity Analysis 15](#_Toc91579678)

[Supplemental Figure 1: Total Payments by Type (All Patients Using Censoring Weights) 17](#_Toc91579683)

[Supplemental Figure 2: Office Visit Utilization Over Time 18](#_Toc91579684)

[Supplemental Figure 3: Hemodialysis Visits Over Time 19](#_Toc91579685)

[Supplemental Figure 4: Hospitalizations Over Time 20](#_Toc91579686)

# Methods

## Study Design

This was a retrospective observational cohort study using data from the USRDS, which collects information about all ESKD patients in the US.^7^ These data included Medicare claims for all fee-for-service medical care received by dialysis patients as well as ancillary data collected as part of the ESKD program and additional data provided by the USRDS coordinating center. Specific details of the files used in these analyses are available in the USRDS Researcher’s Guide.^8^ This research was determined to be exempt from IRB review by Advarra on 02 April 2021.

## Medicare Coverage

The Medicare program provides insurance that helps pay for healthcare services for people with ESKD. The fee-for-service Medicare program provides Part A insurance that covers inpatient hospital care, and Part B insurance that covers provider services and infused medications. Insurance covering oral medications is available (Part D) but not required. In addition, some contracted amounts are paid by patients in the form of a copayment, coinsurance, or deductible. Furthermore, certain services are bundled as part of the provision of dialysis services and are not billed separately. As a result, costs must be estimated across these different sources of payments, and the utilization of services may change over time as services are incorporated into the dialysis bundle.

## Study Observation Period

The USRDS data spanned from January 1, 2015 through December 31, 2018, which defined the largest possible observation period for any patient. For the parathyroidectomy rate analyses, the study index date was the first day of the year for 2016, 2017, and 2018. The baseline period for the parathyroidectomy rate estimation was the 3-month period prior to January 1 of the year for each annual cohort. The follow-up period for parathyroidectomy rate estimation ended on December 31 of each year. Patient follow-up was truncated at the following events: end of continuous Medicare Part A, B, and D enrollment, kidney transplant, parathyroidectomy, death, or the end of the data for the year (i.e., December 31).

For the parathyroidectomy cost and outcomes analyses, the baseline period was the 12-month period prior to parathyroidectomy which could go back as far as January 1, 2015. This period was used to identify inclusion and exclusion criteria as well as other baseline characteristics of the cohort, including dialysis modality. The follow-up period for parathyroidectomy cost estimation was the 12-month period starting on the day of parathyroidectomy. Note that, for patients who received a parathyroidectomy after the day of admission, the day of hospital admission was used as the start of follow-up. Patient follow-up was truncated at the following events: end of continuous Medicare Part A, B, and D enrollment, kidney transplant, death, or the end of the data (December 31, 2018).

## Study Populations

For the parathyroidectomy rate analyses, the study population included all prevalent HD and PD patients age 18 years or older as of the first day of each year from 2016 through 2018. Patients meeting all of the following criteria as of the January 1 index date of each year were included. Patients must have been age 18 years or older and receiving HD or PD based on their dialysis modality as of the start of observation (January 1 of each year). Patients must have been continuously enrolled in Medicare Part A and Part B coverage with Medicare as the primary payer for the prior 3 months (October – December of the prior year). We excluded patients who had a parathyroidectomy during the 3 months prior to the index date of each year.

For the parathyroidectomy cost and outcomes analyses, the study population was all adult HD and PD patients who underwent parathyroidectomy between January 1, 2016 and December 31, 2017 who met the study inclusion and exclusion criteria. ESKD patients meeting the following criteria at the index date (i.e., the parathyroidectomy date) were included. Patients must have been at least 19 years old at the time of parathyroidectomy (to allow for a 12-month lookback period as an adult) and received either HD or PD (but not both) during the 12-month baseline period before parathyroidectomy.

Patients receiving HD must have received at least 21 HD visits over the previous 3 months (i.e., an average of at least 7 of the expected 13 dialysis sessions each month). Since PD is generally done at home, there were no similar requirements for these patients. All patients must have had a minimum time since dialysis initiation (vintage) of at least 12 months. Patients must have had at least 12 months of Medicare Part A, Part B, and Part D coverage with Medicare as the primary payer prior to the date of parathyroidectomy. Finally, the index parathyroidectomy must have been the first parathyroidectomy for the patient according to the available data in the patient’s observation period. We excluded patients who had a diagnosis code for primary hyperparathyroidism in the absence of any diagnosis codes for secondary hyperparathyroidism.

## Baseline Variables

For parathyroidectomy rate estimation, the key baseline variables were age, sex, dialysis modality, and ESKD network to facilitate stratification of rates by these factors. For the cost of parathyroidectomy cohort, the following baseline variables were assessed during the baseline period: age, sex, race, dialysis modality, time since dialysis initiation (vintage), cause of ESKD, and comorbid conditions. Comorbid conditions were based on the Charlson Comorbidity Index, adapted for International Classification of Diseases, 10^th^ Revision, Clinical Modification (ICD-10-CM) codes, which assesses 17 comorbid conditions including myocardial infarction, congestive heart failure, peripheral vascular disease, cerebrovascular disease, chronic pulmonary disease, liver disease, cancer history, and diabetes.^9^ We excluded renal disease from the scoring. Phosphate binder, calcimimetics, and intravenous (IV) vitamin D were identified from Part B and Part D claims. Oral vitamin D was not available for 2015 or 2016 and was not reported.

## Outcome Variables

For the parathyroidectomy rate estimation annual cohorts, the outcome was parathyroidectomy based on the date of service for the procedure.

For the parathyroidectomy cost estimation cohort, we estimated costs, resource utilization, surgical side effects, and laboratory values before and after parathyroidectomy. The cost of care in patients receiving parathyroidectomy was defined as Medicare payment and patient contracted amounts (copayment, coinsurance, and deductible) for all covered services, adjusted to 2020 using the full-year 2020 medical component of the Consumer Price Index. We stratified the total cost by type (inpatient facility, outpatient facility, physician services, Part D oral medications, and all other costs as a separate group).

Resource utilization was also assessed and included dialysis visits, hospitalization, hospitalization days, and office visits. Surgical side effects were based on diagnosis codes for vocal cord paralysis or laryngeal nerve injury, hematoma/bleeding related to surgery, wound infection related to surgery, and seroma formation related to surgery (Supplementary Materials Table 1).^6,10^ Laboratory measures for corrected Ca (cCa) and P were based on CROWNWeb data (PTH was not available). We excluded laboratory values in the month of parathyroidectomy because only the month and year were known and therefore “before” and “after” parathyroidectomy could not be determined. We also identified hypocalcemia (cCa <8.5 mg/dL) and severe hypocalcemia (cCa <7.5 mg/dL) each month.^11,12^ Finally, we identified subsequent parathyroidectomy procedures after the index surgery, and all-cause mortality.

## Analyses

The parathyroidectomy rate was calculated as the number of parathyroidectomy patients in each annual cohort divided by the total person-years of follow-up (in thousands), assuming a Poisson distribution for the variance.^13^ Analyses were stratified by the following factors: modality (HD versus PD), year (2016-2018), vintage group (0-2, 2-5, and 5+ years since dialysis initiation), ESKD network, and race. We also combined the results for all years in a Poisson regression model to estimate the relative contributions of each factor.

Unadjusted cumulative costs were estimated over the 12-month baseline and follow-up periods for the cohort. Costs were partitioned by month and, because patients were censored during the follow-up period, we used inverse probability of censoring weights to account for patients who were lost to follow-up. These estimates are based on the Kaplan-Meier estimator where the roles of censoring and death are switched.^14,15^ Note that death is not a censoring event in a cumulative cost analysis. We estimated the difference in the 12-month cost estimates before and after parathyroidectomy using bootstrapping to estimate 95% confidence intervals. This same approach was used when partitioning the total cost into subtypes.

Resource utilization rates were estimated using the number of events in the numerator, and the person-time of follow-up in the denominator. Differences were estimated between the 12-month rates before and after parathyroidectomy with 95% confidence intervals estimated by bootstrapping. Surgical complication rates were estimated as the difference between the cumulative 90-day rates for the periods before and after parathyroidectomy, with confidence intervals estimated using bootstrapping.

Laboratory data for cCa and P were summarized monthly by averaging all values within each interval and are reported as means. Monthly hypocalcemia events were based on monthly cCa measures, and are summarized as a proportion per month, using the number of people with hypocalcemia in the numerator, and the number of patients with a cCa measure in the denominator. The rate of subsequent parathyroidectomy and the rate of death were estimated per 1,000 person-years.

Analyses related to cost were conducted overall as well as for HD and PD patients separately. We conducted a sensitivity analysis of parathyroidectomy cost using complete cases, defined as patients alive and uncensored through the end of month 12 after parathyroidectomy.

The raw data were decrypted and loaded onto a server. Using R, the raw data were converted to a standardized format (Generalized Data Model^16^) and then moved into a PostgreSQL database. Jigsaw software (Outcomes Insights, Inc., Agoura Hills, CA) was used to create algorithms for extracting data and creating tables of all events of interest. The final analysis dataset was created in R (version 3.6.3)^17^ based on the Jigsaw tables. All statistical analyses were performed using R.

# Tables

Supplemental Table 1: Diagnosis codes for surgical side effects

| **Vocabulary** | **Code** | **Description** |
| --- | --- | --- |
| ***Hematoma or hemorrhage*** | | |
| ICD-9 CM | 998.11 | Hemorrhage complicating a procedure |
| ICD-9 CM | 998.12 | Hematoma complicating a procedure |
| ICD-10 CM | E89.810 | Postprocedural hemorrhage of an endocrine system organ or structure following an endocrine system procedure |
| ICD-10 CM | E89.811 | Postprocedural hemorrhage of an endocrine system organ or structure following other procedure |
| ICD-10 CM | E89.820 | Postprocedural hematoma of an endocrine system organ or structure following an endocrine system procedure |
| ICD-10 CM | E89.821 | Postprocedural hematoma of an endocrine system organ or structure following other procedure |
| ICD-10 CM | L76.22 | Postprocedural hemorrhage of skin and subcutaneous tissue following other procedure |
| ICD-10 CM | L76.32 | Postprocedural hematoma of skin and subcutaneous tissue following other procedure |
| ***Seroma*** | | |
| ICD-9 CM | 998.13 | Seroma complicating a procedure |
| ICD-9 CM | 998.51 | Infected postoperative seroma |
| ICD-10 CM | E89.822 | Postprocedural seroma of an endocrine system organ or structure following an endocrine system procedure |
| ICD-10 CM | E89.823 | Postprocedural seroma of an endocrine system organ or structure following other procedure |
| ICD-10 CM | L76.34 | Postprocedural seroma of skin and subcutaneous tissue following other procedure |
| ***Wound infection*** | | |
| ICD-9 CM | 998.51 | Infected postoperative seroma |
| ICD-9 CM | 998.59 | Other postoperative infection |
| ICD-10 CM | T81.40XA | Infection following a procedure, unspecified, initial encounter |
| ICD-10 CM | T81.40XD | Infection following a procedure, unspecified, subsequent encounter |
| ICD-10 CM | T81.40XS | Infection following a procedure, unspecified, sequela |
| ICD-10 CM | T81.41XA | Infection following a procedure, superficial incisional surgical site, initial encounter |
| ICD-10 CM | T81.41XD | Infection following a procedure, superficial incisional surgical site, subsequent encounter |
| ICD-10 CM | T81.41XS | Infection following a procedure, superficial incisional surgical site, sequela |
| ICD-10 CM | T81.42XA | Infection following a procedure, deep incisional surgical site, initial encounter |
| ICD-10 CM | T81.42XD | Infection following a procedure, deep incisional surgical site, subsequent encounter |
| ICD-10 CM | T81.42XS | Infection following a procedure, deep incisional surgical site, sequela |
| ICD-10 CM | T81.43XA | Infection following a procedure, organ and space surgical site, initial encounter |
| ICD-10 CM | T81.43XD | Infection following a procedure, organ and space surgical site, subsequent encounter |
| ICD-10 CM | T81.43XS | Infection following a procedure, organ and space surgical site, sequela |
| ICD-10 CM | T81.49XA | Infection following a procedure, other surgical site, initial encounter |
| ICD-10 CM | T81.49XD | Infection following a procedure, other surgical site, subsequent encounter |
| ICD-10 CM | T81.49XS | Infection following a procedure, other surgical site, sequela |
| ***Vocal cord paralysis*** | | |
| ICD-9 CM | 478.3 | Paralysis of vocal cords or larynx, unspecified |
| ICD-9 CM | 478.31 | Unilateral paralysis of vocal cords or larynx, partial |
| ICD-9 CM | 478.32 | Unilateral paralysis of vocal cords or larynx, complete |
| ICD-9 CM | 478.33 | Bilateral paralysis of vocal cords or larynx, partial |
| ICD-9 CM | 478.34 | Bilateral paralysis of vocal cords or larynx, complete |
| ICD-10 CM | J38.00 | Paralysis of vocal cords and larynx, unspecified |
| ICD-10 CM | J38.01 | Paralysis of vocal cords and larynx, unilateral |
| ICD-10 CM | J38.02 | Paralysis of vocal cords and larynx, bilateral |

Supplemental Table 2: Rate of Parathyroidectomy Overall and Within Strata: 2016

| Subgroup | Stratum | Patients | Events | Person Years | Rate per 1,000 | SE | 95% CI |
| --- | --- | --- | --- | --- | --- | --- | --- |
| Overall | Overall | 303,665 | 1,764 | 271,471 | 6.5 | 0.2 | 6.2 - 6.8 |
| Modality | HD | 276,358 | 1,508 | 246,878 | 6.1 | 0.2 | 5.8 - 6.4 |
| Modality | PD | 27,307 | 256 | 24,594 | 10.4 | 0.7 | 9.1 - 11.7 |
| Age Group | <40 years | 20,885 | 424 | 19,391 | 21.9 | 1.1 | 19.8 - 23.9 |
| Age Group | 40-49 years | 34,294 | 430 | 31,886 | 13.5 | 0.7 | 12.2 - 14.8 |
| Age Group | 50-59 years | 62,811 | 497 | 57,804 | 8.6 | 0.4 | 7.8 - 9.4 |
| Age Group | 60-69 years | 83,312 | 289 | 74,817 | 3.9 | 0.2 | 3.4 - 4.3 |
| Age Group | 70-79 years | 64,970 | 103 | 56,599 | 1.8 | 0.2 | 1.5 - 2.2 |
| Age Group | 80+ years | 37,393 | 21 | 30,973 | 0.7 | 0.1 | 0.4 - 1.0 |
| Race | White | 169,586 | 749 | 149,050 | 5.0 | 0.2 | 4.7 - 5.4 |
| Race | Black | 113,452 | 929 | 103,651 | 9.0 | 0.3 | 8.4 - 9.5 |
| Race | Asian | 12,127 | 45 | 11,019 | 4.1 | 0.6 | 2.9 - 5.3 |
| Race | All Other | 8,500 | 41 | 7,751 | 5.3 | 0.8 | 3.7 - 6.9 |
| Network | 01 | 9,573 | 68 | 8,442 | 8.1 | 1.0 | 6.1 - 10.0 |
| Network | 02 | 16,945 | 65 | 15,015 | 4.3 | 0.5 | 3.3 - 5.4 |
| Network | 03 | 12,108 | 47 | 10,809 | 4.3 | 0.6 | 3.1 - 5.6 |
| Network | 04 | 11,660 | 54 | 10,315 | 5.2 | 0.7 | 3.8 - 6.6 |
| Network | 05 | 18,960 | 90 | 16,971 | 5.3 | 0.6 | 4.2 - 6.4 |
| Network | 06 | 32,598 | 282 | 29,535 | 9.5 | 0.6 | 8.4 - 10.7 |
| Network | 07 | 18,835 | 101 | 16,725 | 6.0 | 0.6 | 4.9 - 7.2 |
| Network | 08 | 18,966 | 194 | 17,015 | 11.4 | 0.8 | 9.8 - 13.0 |
| Network | 09 | 20,037 | 84 | 17,631 | 4.8 | 0.5 | 3.7 - 5.8 |
| Network | 10 | 12,437 | 52 | 11,078 | 4.7 | 0.7 | 3.4 - 6.0 |
| Network | 11 | 18,211 | 91 | 16,097 | 5.7 | 0.6 | 4.5 - 6.8 |
| Network | 12 | 11,025 | 81 | 9,730 | 8.3 | 0.9 | 6.5 - 10.1 |
| Network | 13 | 13,398 | 101 | 12,008 | 8.4 | 0.8 | 6.8 - 10.1 |
| Network | 14 | 30,890 | 183 | 27,816 | 6.6 | 0.5 | 5.6 - 7.5 |
| Network | 15 | 14,125 | 77 | 12,670 | 6.1 | 0.7 | 4.7 - 7.4 |
| Network | 16 | 8,719 | 53 | 7,749 | 6.8 | 0.9 | 5.0 - 8.7 |
| Network | 17 | 14,078 | 63 | 12,748 | 4.9 | 0.6 | 3.7 - 6.2 |
| Network | 18 | 21,100 | 78 | 19,116 | 4.1 | 0.5 | 3.2 - 5.0 |
| Vintage Group | 0-2 years | 87,061 | 162 | 78,010 | 2.1 | 0.2 | 1.8 - 2.4 |
| Vintage Group | 2-5 years | 101,561 | 539 | 90,813 | 5.9 | 0.3 | 5.4 - 6.4 |
| Vintage Group | 5+ years | 115,043 | 1,063 | 102,648 | 10.4 | 0.3 | 9.7 - 11.0 |

Rates are per 1,000 person-years. NR = counts < 11 not reportable according to the data use agreement with the USRDS.

Supplemental Table 3: Rate of Parathyroidectomy Overall and Within Strata: 2017

| Subgroup | Stratum | Patients | Events | Person Years | Rate per 1,000 | SE | 95% CI |
| --- | --- | --- | --- | --- | --- | --- | --- |
| Overall | Overall | 307,986 | 1,598 | 273,822 | 5.8 | 0.1 | 5.5 - 6.1 |
| Modality | HD | 279,985 | 1,379 | 248,839 | 5.5 | 0.1 | 5.2 - 5.8 |
| Modality | PD | 28,001 | 219 | 24,983 | 8.8 | 0.6 | 7.6 - 9.9 |
| Age Group | <40 years | 20,615 | 367 | 19,034 | 19.3 | 1.0 | 17.3 - 21.3 |
| Age Group | 40-49 years | 33,761 | 377 | 31,254 | 12.1 | 0.6 | 10.8 - 13.3 |
| Age Group | 50-59 years | 62,666 | 446 | 57,372 | 7.8 | 0.4 | 7.1 - 8.5 |
| Age Group | 60-69 years | 85,998 | 307 | 76,839 | 4.0 | 0.2 | 3.5 - 4.4 |
| Age Group | 70-79 years | 67,085 | 93 | 58,220 | 1.6 | 0.2 | 1.3 - 1.9 |
| Age Group | 80+ years | 37,861 | NR | NR | 0.3 | 0.1 | 0.1 - 0.4 |
| Race | White | 173,424 | 676 | 151,720 | 4.5 | 0.2 | 4.1 - 4.8 |
| Race | Black | 113,071 | 852 | 102,699 | 8.3 | 0.3 | 7.7 - 8.9 |
| Race | Asian | 12,679 | 34 | 11,396 | 3.0 | 0.5 | 2.0 - 4.0 |
| Race | All Other | 8,812 | 36 | 8,006 | 4.5 | 0.7 | 3.0 - 6.0 |
| Network | 01 | 9,764 | 63 | 8,586 | 7.3 | 0.9 | 5.5 - 9.1 |
| Network | 02 | 17,068 | 86 | 15,092 | 5.7 | 0.6 | 4.5 - 6.9 |
| Network | 03 | 12,141 | 38 | 10,808 | 3.5 | 0.6 | 2.4 - 4.6 |
| Network | 04 | 11,689 | 69 | 10,312 | 6.7 | 0.8 | 5.1 - 8.3 |
| Network | 05 | 19,121 | 69 | 16,977 | 4.1 | 0.5 | 3.1 - 5.0 |
| Network | 06 | 33,035 | 263 | 29,730 | 8.8 | 0.5 | 7.8 - 9.9 |
| Network | 07 | 19,143 | 90 | 16,898 | 5.3 | 0.6 | 4.2 - 6.4 |
| Network | 08 | 19,194 | 130 | 17,109 | 7.6 | 0.7 | 6.3 - 8.9 |
| Network | 09 | 20,295 | 93 | 17,757 | 5.2 | 0.5 | 4.2 - 6.3 |
| Network | 10 | 12,958 | 52 | 11,461 | 4.5 | 0.6 | 3.3 - 5.8 |
| Network | 11 | 18,382 | 81 | 16,212 | 5.0 | 0.6 | 3.9 - 6.1 |
| Network | 12 | 11,076 | 87 | 9,726 | 8.9 | 1.0 | 7.1 - 10.8 |
| Network | 13 | 13,737 | 74 | 12,176 | 6.1 | 0.7 | 4.7 - 7.5 |
| Network | 14 | 31,557 | 174 | 28,307 | 6.1 | 0.5 | 5.2 - 7.1 |
| Network | 15 | 14,249 | 59 | 12,704 | 4.6 | 0.6 | 3.5 - 5.8 |
| Network | 16 | 8,907 | 47 | 7,896 | 6.0 | 0.9 | 4.3 - 7.7 |
| Network | 17 | 14,357 | 56 | 12,903 | 4.3 | 0.6 | 3.2 - 5.5 |
| Network | 18 | 21,313 | 67 | 19,168 | 3.5 | 0.4 | 2.7 - 4.3 |
| Vintage Group | 0-2 years | 87,650 | 122 | 78,095 | 1.6 | 0.1 | 1.3 - 1.8 |
| Vintage Group | 2-5 years | 102,888 | 472 | 91,555 | 5.2 | 0.2 | 4.7 - 5.6 |
| Vintage Group | 5+ years | 117,448 | 1,004 | 104,171 | 9.6 | 0.3 | 9.0 - 10.2 |

Rates are per 1,000 person-years. NR = counts < 11 not reportable according to the data use agreement with the USRDS.

Supplemental Table 4: Rate of Parathyroidectomy Overall and Within Strata: 2018

| Subgroup | Stratum | Patients | Events | Person Years | Rate per 1,000 | SE | 95% CI |
| --- | --- | --- | --- | --- | --- | --- | --- |
| Overall | Overall | 303,729 | 1,435 | 269,815 | 5.3 | 0.1 | 5.0 - 5.6 |
| Modality | HD | 276,667 | 1,214 | 245,741 | 4.9 | 0.1 | 4.7 - 5.2 |
| Modality | PD | 27,062 | 221 | 24,074 | 9.2 | 0.6 | 8.0 - 10.4 |
| Age Group | <40 years | 19,766 | 364 | 18,206 | 20.0 | 1.0 | 17.9 - 22.0 |
| Age Group | 40-49 years | 32,840 | 359 | 30,379 | 11.8 | 0.6 | 10.6 - 13.0 |
| Age Group | 50-59 years | 60,909 | 381 | 55,661 | 6.8 | 0.4 | 6.2 - 7.5 |
| Age Group | 60-69 years | 84,990 | 239 | 75,896 | 3.1 | 0.2 | 2.7 - 3.5 |
| Age Group | 70-79 years | 68,005 | 82 | 59,015 | 1.4 | 0.2 | 1.1 - 1.7 |
| Age Group | 80+ years | 37,219 | NR | NR | 0.3 | 0.1 | 0.1 - 0.5 |
| Race | White | 172,646 | 614 | 151,010 | 4.1 | 0.2 | 3.7 - 4.4 |
| Race | Black | 109,533 | 763 | 99,305 | 7.7 | 0.3 | 7.1 - 8.2 |
| Race | Asian | 12,693 | 30 | 11,420 | 2.6 | 0.5 | 1.7 - 3.6 |
| Race | All Other | 8,857 | 28 | 8,079 | 3.5 | 0.7 | 2.2 - 4.7 |
| Network | 01 | 9,582 | 48 | 8,412 | 5.7 | 0.8 | 4.1 - 7.3 |
| Network | 02 | 17,073 | 67 | 15,019 | 4.5 | 0.5 | 3.4 - 5.5 |
| Network | 03 | 11,824 | 36 | 10,526 | 3.4 | 0.6 | 2.3 - 4.5 |
| Network | 04 | 11,653 | 67 | 10,263 | 6.5 | 0.8 | 5.0 - 8.1 |
| Network | 05 | 18,828 | 51 | 16,720 | 3.1 | 0.4 | 2.2 - 3.9 |
| Network | 06 | 32,280 | 242 | 29,036 | 8.3 | 0.5 | 7.3 - 9.4 |
| Network | 07 | 18,973 | 77 | 16,750 | 4.6 | 0.5 | 3.6 - 5.6 |
| Network | 08 | 18,590 | 155 | 16,511 | 9.4 | 0.8 | 7.9 - 10.9 |
| Network | 09 | 19,688 | 88 | 17,222 | 5.1 | 0.5 | 4.0 - 6.2 |
| Network | 10 | 12,515 | 53 | 11,100 | 4.8 | 0.7 | 3.5 - 6.1 |
| Network | 11 | 18,410 | 69 | 16,231 | 4.3 | 0.5 | 3.2 - 5.3 |
| Network | 12 | 11,114 | 60 | 9,708 | 6.2 | 0.8 | 4.6 - 7.7 |
| Network | 13 | 13,682 | 82 | 12,172 | 6.7 | 0.7 | 5.3 - 8.2 |
| Network | 14 | 31,022 | 130 | 27,786 | 4.7 | 0.4 | 3.9 - 5.5 |
| Network | 15 | 14,287 | 82 | 12,729 | 6.4 | 0.7 | 5.0 - 7.8 |
| Network | 16 | 8,939 | 45 | 7,940 | 5.7 | 0.8 | 4.0 - 7.3 |
| Network | 17 | 14,380 | 30 | 12,975 | 2.3 | 0.4 | 1.5 - 3.1 |
| Network | 18 | 20,889 | 53 | 18,714 | 2.8 | 0.4 | 2.1 - 3.6 |
| Vintage Group | 0-2 years | 85,019 | 118 | 75,893 | 1.6 | 0.1 | 1.3 - 1.8 |
| Vintage Group | 2-5 years | 102,438 | 429 | 90,997 | 4.7 | 0.2 | 4.3 - 5.2 |
| Vintage Group | 5+ years | 116,272 | 888 | 102,924 | 8.6 | 0.3 | 8.1 - 9.2 |

Rates are per 1,000 person-years. NR = counts < 11 not reportable according to the data use agreement with the USRDS.

Supplemental Table 5: Poisson Regression Model of Factors Associated with Parathyroidectomy

| Variable | Relative Rate | 95% CI | P-value |
| --- | --- | --- | --- |
| PD (vs. HD) | 1.70 | 1.56 - 1.84 | <0.0001 |
| Age 40-49 (vs. < 40 years) | 0.61 | 0.56 - 0.66 | <0.0001 |
| Age 50-59 (vs. < 40 years) | 0.40 | 0.37 - 0.43 | <0.0001 |
| Age 60-69 (vs. < 40 years) | 0.20 | 0.19 - 0.22 | <0.0001 |
| Age 70-79 (vs. < 40 years) | 0.10 | 0.09 - 0.11 | <0.0001 |
| Age 80+ (vs. < 40 years) | 0.03 | 0.02 - 0.04 | <0.0001 |
| Vintage 2-5 years (vs. < 2 years) | 2.69 | 2.41 - 3.01 | <0.0001 |
| Vintage 5+ years (vs. < 2 years) | 4.38 | 3.94 - 4.86 | <0.0001 |
| Black (vs. White) | 1.33 | 1.25 - 1.41 | <0.0001 |
| Asian (vs. White) | 0.71 | 0.58 - 0.86 | 0.0005 |
| All Other Race (vs. White) | 0.77 | 0.63 - 0.93 | 0.0084 |
| 2017 (vs. 2016) | 0.91 | 0.85 - 0.97 | 0.0055 |
| 2018 (vs. 2016) | 0.84 | 0.78 - 0.90 | <0.0001 |

Results from pooling the 2016, 2017, and 2018 cohorts. Intercept term not shown.

Supplemental Table 6: Parathyroidectomy Cohort Attrition Table

| Cohort Processing Step | Qualified | Available | Percent Qualified |
| --- | --- | --- | --- |
| Total Population (any patient with PTx code) | 11014 |  |  |
| Met enrollment criteria (Medicare Parts A, B, & D no HMO) within export observation date range [1964-11-10 - 2022-11-02] | 11003 | 11014 | 99.90 |
| Has index event in enrollment window | 10939 | 11003 | 99.42 |
| Has 'Parathyroidectomy' | 10939 | 10939 | 100.00 |
| Has index event between study date range of [2016-01-01 - 2017-12-31] | 5686 | 10939 | 51.98 |
| Met specified age ranges: [19, 200) | 5673 | 5686 | 99.77 |
| Has enrollment period with sufficient minimum lookback and follow up periods | 5487 | 5673 | 96.72 |
| Has AT LEAST 1 of 2 events in baseline period | 4679 | 5487 | 85.27 |
| Has 'Dialysis Modality: Hemodialysis' event in baseline period | 4219 | 5487 | 76.89 |
| Has 'Dialysis Modality: Peritoneal Dialysis' event in baseline period | 747 | 5487 | 13.61 |
| Has NONE of the exclusion events in baseline period | 3937 | 4679 | 84.14 |
| Has 'Date of Dialysis Start' | 0 | 4679 | 0.00 |
| Has 'Home hemodialysis, center self-hemodialysis, or other peritoneal dialysis' | 300 | 4679 | 6.41 |
| Has 'USRDS Kidney Transplant' | 193 | 4679 | 4.12 |
| Has 'Hemodialysis and peritoneal dialysis' | 287 | 4679 | 6.13 |
| Has 'death record during baseline' | NR | 4679 | NR |
| Additional Criteria Applied to Cohort |  |  |  |
| Patients Without History of Transplant | 3255 | 3937 | 82.68 |
| HD patients with >= 21 dialysis visits in last 3 months | 3157 | 3255 | 96.99 |
| No PHPT diagnosis | 3155 | 3157 | 99.94 |
| Has modality records during baseline period | 3008 | 3155 | 95.34 |
| Final Cohort Population | 3008 | 3008 | 100.00 |

NR = counts < 11 not reportable according to the data use agreement with the USRDS.

Supplemental Table 7: Costs Before and After Parathyroidectomy, Overall and by Dialysis Modality: Sensitivity Analysis of Patients Who Were Alive and Observable at 12 Months After Parathyroidectomy

| **Cost Type** | **Before** | **After** | **Difference** | **Lower CI** | **Upper CI** |
| --- | --- | --- | --- | --- | --- |
| ***Overall*** | | | | | |
| Inpatient Facility | $17,553 | $41,082 | $23,528 | $21,937 | $24,951 |
| Outpatient Facility | $38,772 | $39,416 | $644 | $311 | $978 |
| Physician/Provider | $12,918 | $14,829 | $1,911 | $1,378 | $2,497 |
| Prescription | $375 | $190 | -$185 | -$210 | -$161 |
| All Other | $893 | $1,316 | $423 | $317 | $531 |
| Total | $70,512 | $96,833 | $26,321 | $24,436 | $28,137 |
| ***Hemodialysis (HD)*** | | | | | |
| Inpatient Facility | $18,787 | $41,317 | $22,530 | $20,779 | $24,083 |
| Outpatient Facility | $39,074 | $39,669 | $595 | $263 | $957 |
| Physician/Provider | $13,537 | $15,100 | $1,563 | $1,046 | $2,164 |
| Prescription | $377 | $187 | -$190 | -$216 | -$164 |
| All Other | $965 | $1,348 | $383 | $267 | $495 |
| Total | $72,740 | $97,621 | $24,881 | $22,922 | $26,780 |
| ***Peritoneal Dialysis (PD)*** | | | | | |
| Inpatient Facility | $3,835 | $38,462 | $34,627 | $28,887 | $39,539 |
| Outpatient Facility | $35,423 | $36,604 | $1,182 | $177 | $2,126 |
| Physician/Provider | $6,038 | $11,822 | $5,784 | $4,231 | $7,135 |
| Prescription | $353 | $219 | -$134 | -$218 | -$46 |
| All Other | $95 | $968 | $873 | $557 | $1,159 |
| Total | $45,744 | $88,075 | $42,331 | $35,587 | $48,247 |

Note: Only patients who were alive and observable at 12 months after parathyroidectomy were included (N = 2,581). Costs include all payer and patient paid amounts. Prescription costs include only Medicare Part D costs. All costs are inflated to 2020 dollars. Confidence intervals estimated using bootstrapping. “Before” and “After” reflect the mean cost for the 12-month periods before and after parathyroidectomy. The cost of parathyroidectomy is included in the “after” interval. HD = hemodialysis, PD = peritoneal dialysis.

# Figures

Supplemental Figure 1: Total Payments by Type (All Patients Using Censoring Weights)

Supplemental Figure 2: Office Visit Utilization Over Time


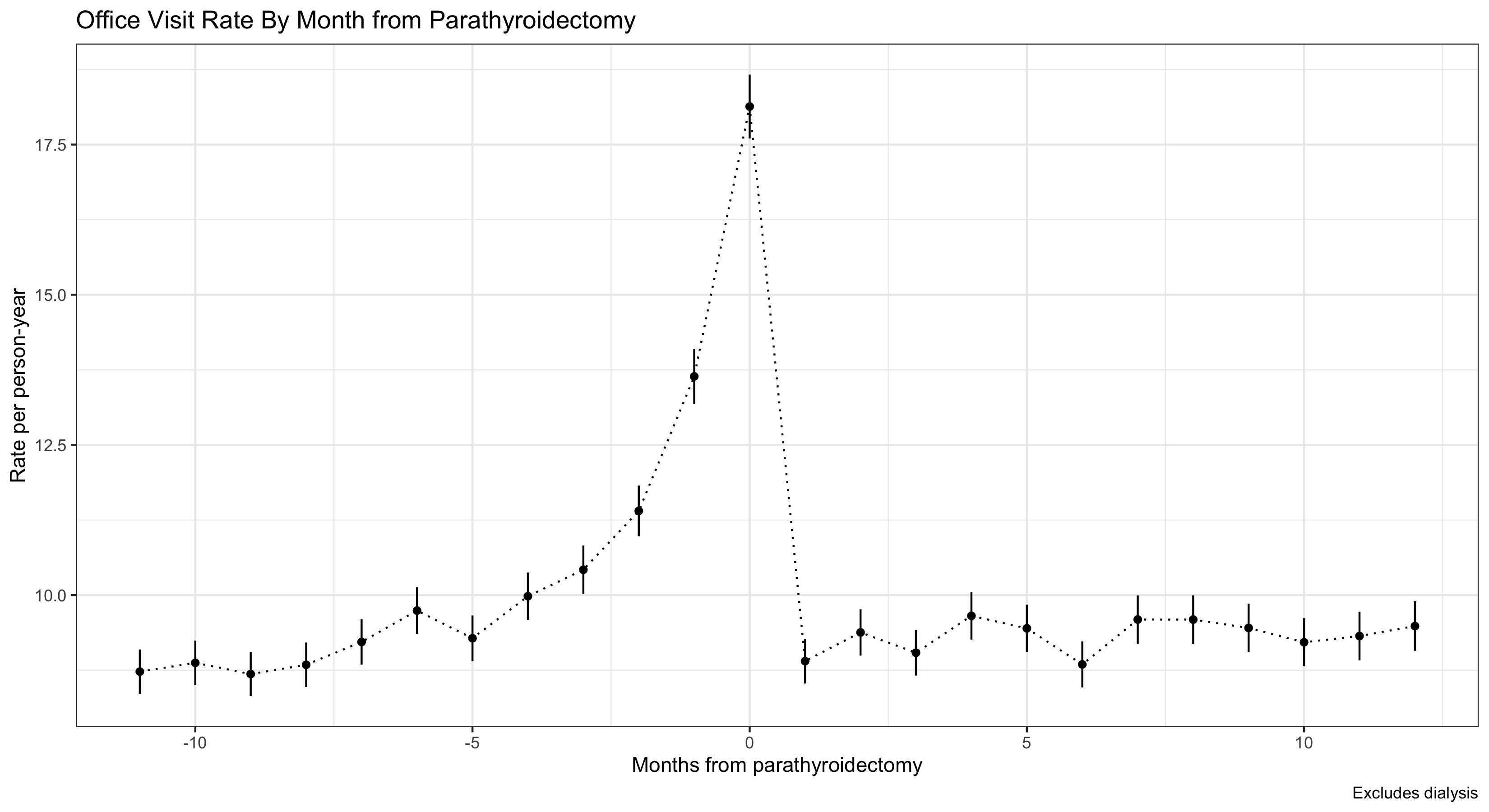


Parathyroidectomy occurred in month 1. Month 0 ends the day before parathyroidectomy event. Dialysis visits are excluded.

Supplemental Figure 3: Hemodialysis Visits Over Time


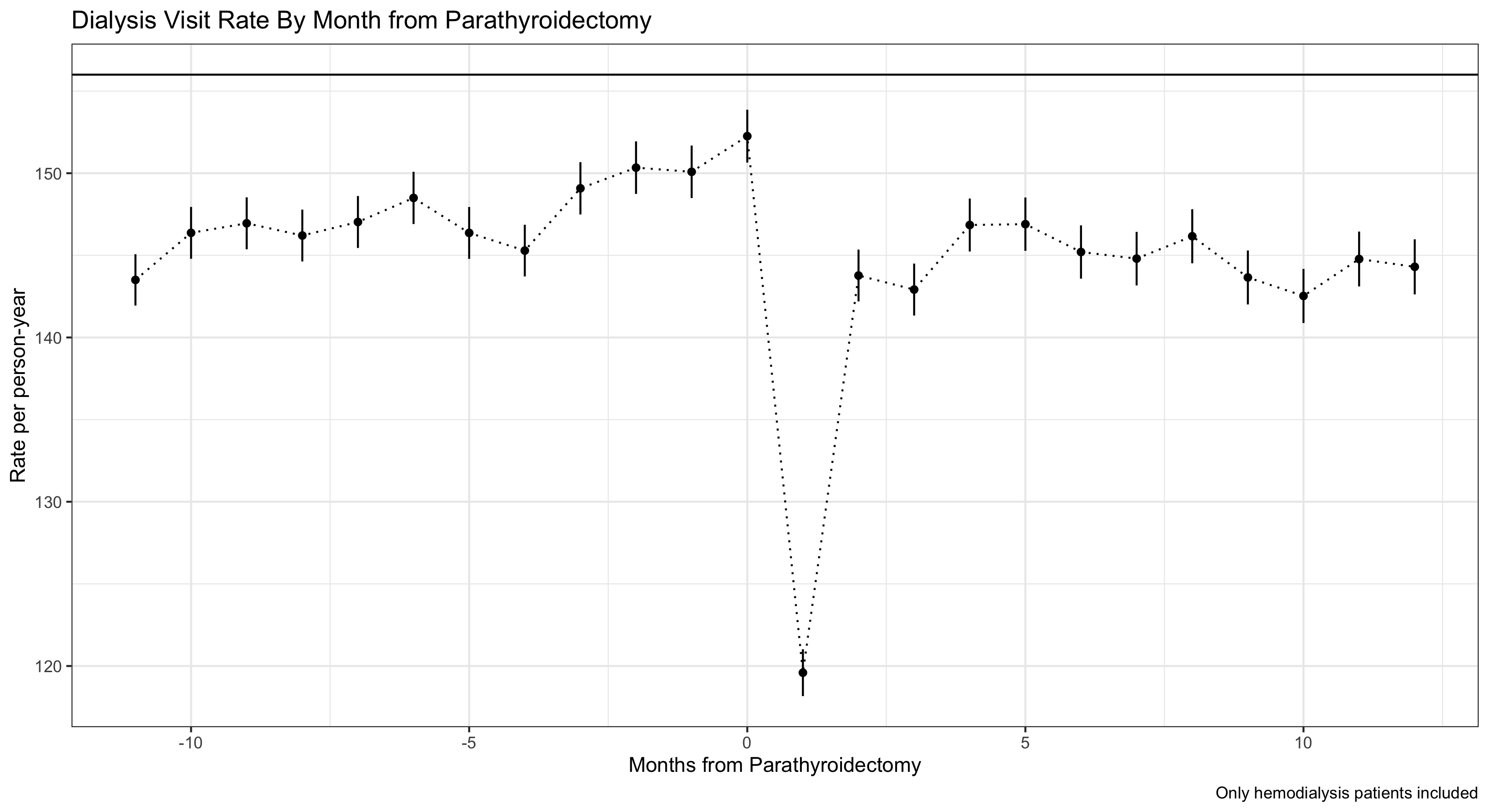


Parathyroidectomy occurred in month 1. Month 0 ends the day before parathyroidectomy event. Black horizontal line represents 156 visits which is the approximate expected number of dialysis visits in a year (52 weeks x 3 dialysis sessions per week).

Supplemental Figure 4: Hospitalizations Over Time


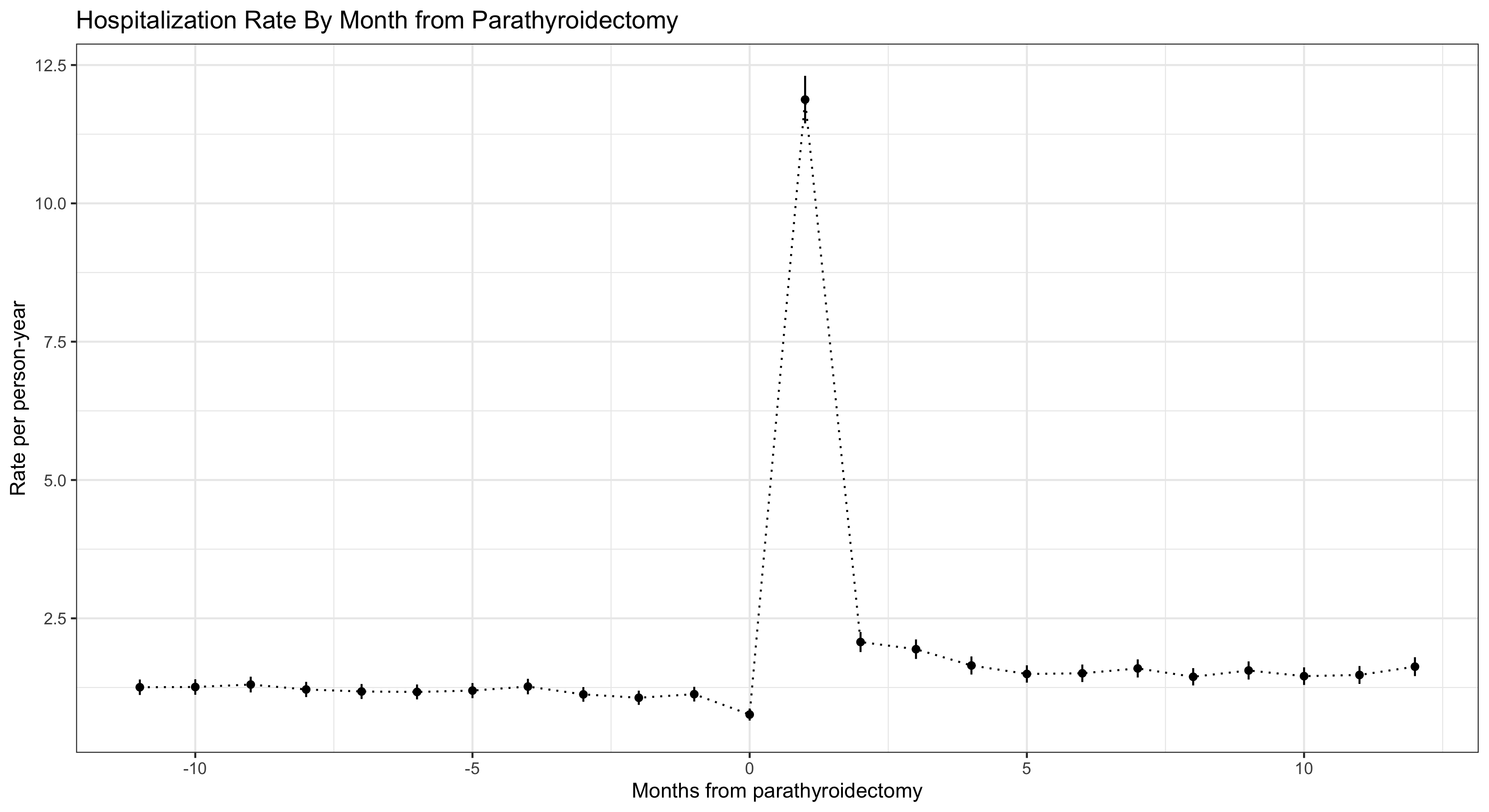


Parathyroidectomy occurred in month 1. Month 0 ends the day before parathyroidectomy event. Hospitalizations are assigned to intervals using the admission date.
